# Multiple pilomatricomas in twins with Rubinstein-Taybi syndrome^☆^^☆☆^

**DOI:** 10.1016/j.abd.2020.03.011

**Published:** 2020-07-15

**Authors:** Ana Laura Andrade Bueno, Maria Emilia Vieira de Souza, Carla Graziadio, Ana Elisa Kiszewski

**Affiliations:** a Dermatology Service, Universidade Federal de Ciências da Saúde de Porto Alegre, Porto Alegre, RS, Brazil; b Irmandade da Santa Casa de Misericórdia de Porto Alegre, Porto Alegre, RS, Brazil; c Discipline of Clinical Genetics, Universidade Federal de Ciências da Saúde de Porto Alegre, Porto Alegre, RS, Brazil; d Pediatric Dermatology Unit, Dermatology Service, Universidade Federal de Ciências da Saúde de Porto Alegre, Porto Alegre, RS, Brazil

**Keywords:** Pilomatrixoma, Pilomatricoma, Rubinstein-Taybi syndrome, Tumor of the skin appendages

## Abstract

Pilomatricomas are benign tumors originating from the capillary matrix, which may present as solitary lesions or, less commonly, multiple. Myotonic dystrophy and familial adenomatous polyposis are the most frequently associated disorders with multiple pilomatricomas. There are few reports relating these tumors to other genetic syndromes. Rubinstein-Taybi syndrome is a rare autosomal dominant disorder characterized by intellectual disability and typical dysmorphic characteristics. There are five case reports relating to multiple pilomatricoma to Rubinstein-Taybi syndrome, an association that needs to be clarified. For this reason, we report the first case of multiple pilomatricoma in monozygotic twins with typical Rubinstein-Taybi syndrome.

## Introduction

Pilomatricomas are uncommon benign tumors derived from hair matrix. They mainly affect the pediatric population and are more frequently located on the head and neck. They present clinically as nodules with firm or stony consistency, circumscribed, normochromic, or erythematous, which can be confused with epidermal cysts. Although they usually present as solitary lesions, multiple pilomatricomas can be observed in 2.4% to 5% of cases.^1^, ^2^, ^3^

Multiple pilomatricomas can be sporadic, familial, or associated with an underlying syndrome. Myotonic dystrophy and familial adenomatous polyposis (FAP) are the most frequently associated disorders with multiple pilomatricomas. There are sporadic reports of the association of these tumors with Turner, Kabuki, Sotos, and Rubinstein-Taybi syndromes.^2^

Rubinstein-Taybi Syndrome (RTS) is a rare autosomal dominant genetic disorder, characterized by postnatal growth retardation, moderate to severe intellectual disability, and a wide range of typical dysmorphic characteristics. Broad, angled thumbs and halluces are a distinguishing feature of the syndrome. Facial anomalies include slanted eyelid slits, high and elongated nasal pyramid, and micrognathia. Cardiac malformations, dental alterations, and cryptorchidism are common.^4^ The most common dermatological findings include hemangiomas, hypertrichosis, brachyonychia, and a tendency to keloid formation.^1^

Although approximately 60% of cases are associated with mutations in the CREBBP or EP300 genes, the etiology of RTS is heterogeneous and poorly defined.^1^, ^5^

There are few cases describing the association of pilomatricomas with RTS, and it is not clear whether this association is due to chance.^6^ According to a recent review of the literature, nine cases have been reported thus far, and five of them presented multiple lesions.^2^ The authors report the first case of multiple pilomatricomas in monozygotic twins with typical RTS.

## Case reports

**Figure 1:**
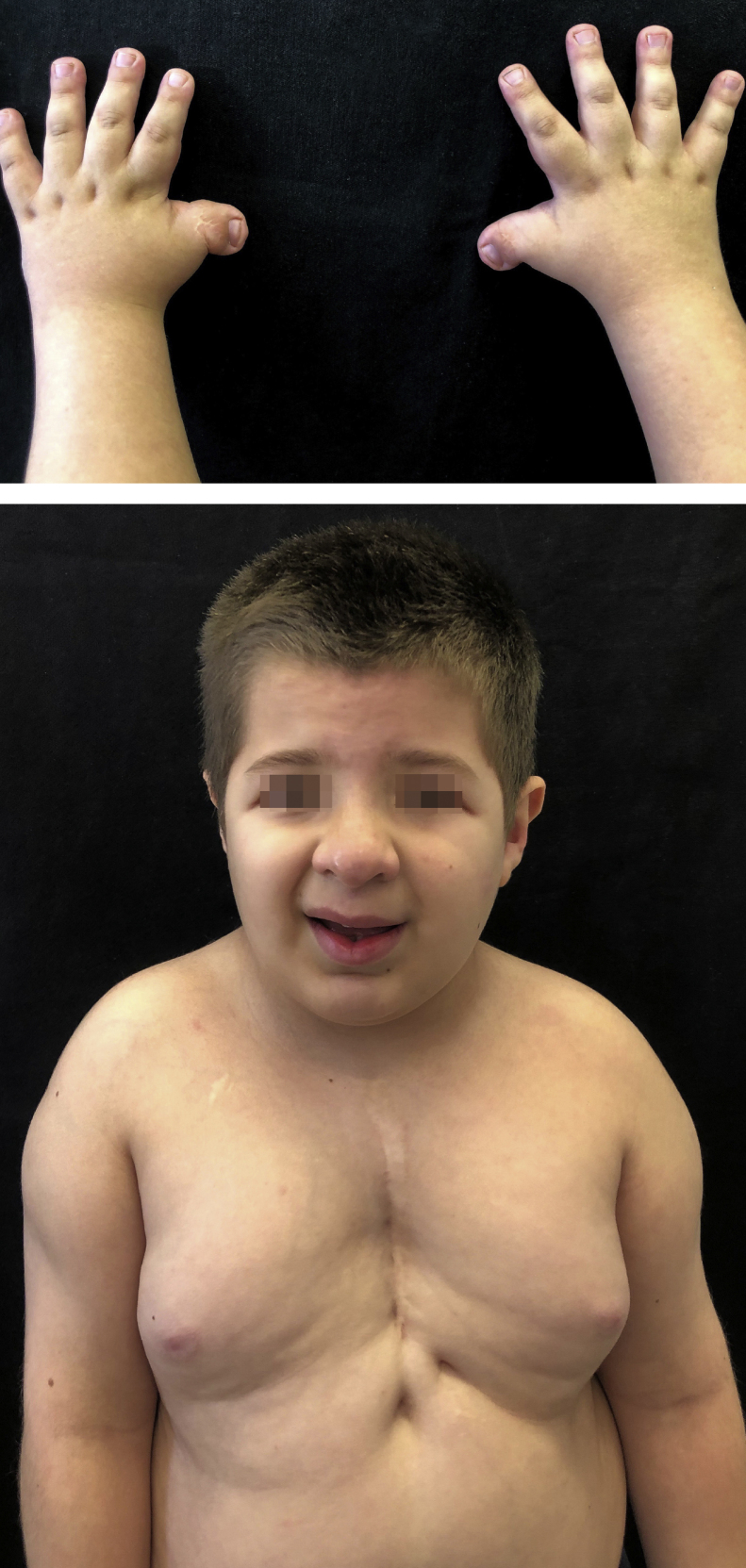
Twin 1 with slanting eyelid slits, discrete micrognathia, prominent auricular helices, nipple hypertelorism, sternal scar secondary to cardiac surgery to correct interventricular communication, wide thumbs and radial deviation.

Monozygotic twins, 8 years old, with delayed neuropsychomotor development, oblique eyelid clefts, discrete micrognathia, ogival palate, prominent auricular helix, nipple hypertelorism, and mild hypertrichosis on the dorsal spine and shoulders. Both had a previous history of cryptorchidism and of short, wide thumbs, whose radial deviation had been surgically corrected. Twin 1 had undergone cardiac surgery to correct interventricular communication and had a previous history of occipital, frontal, and supramammary hemangiomas, which had spontaneously regressed (Fig. 1). Twin 2 presented cerebral aqueduct stenosis and polydactyly, also previously corrected. The karyotype examination of both patients was normal (46, XY) and, in light of the typical phenotypic findings, they were diagnosed with RTS by the genetics team.

**Figure 2:**
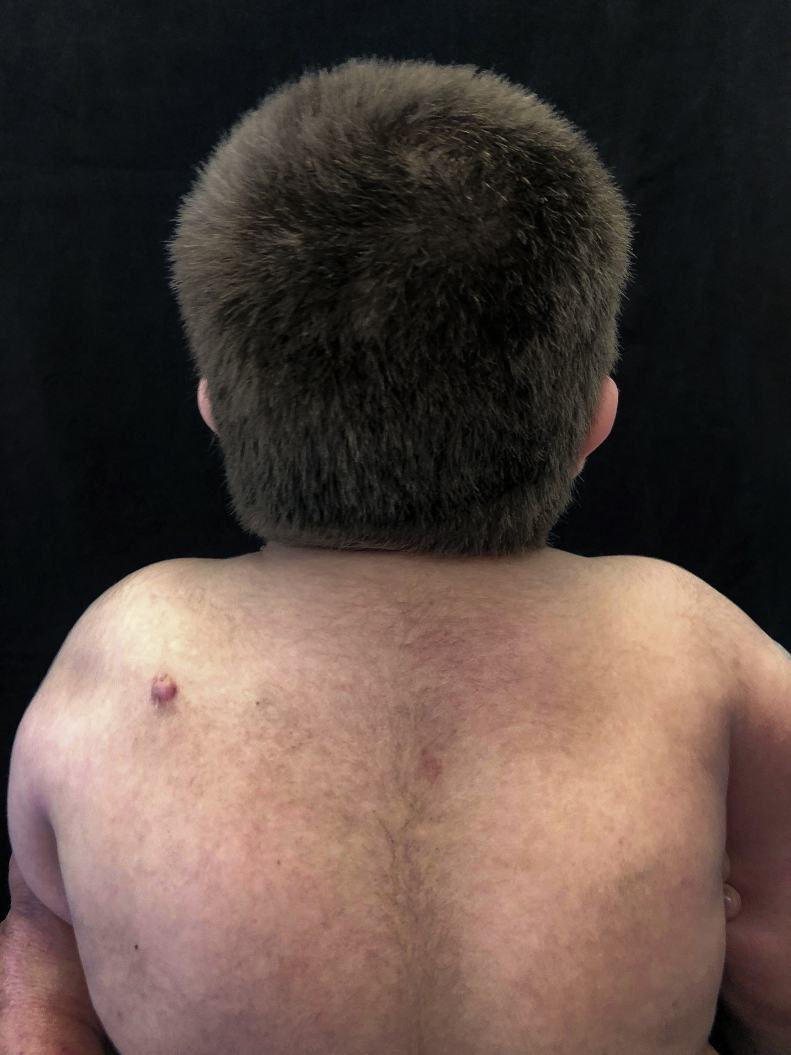
Normochromic nodular lesion in the left scapular region which was excised. Mild hypertrichosis on the dorsal spine and shoulders.

**Figure 3:**
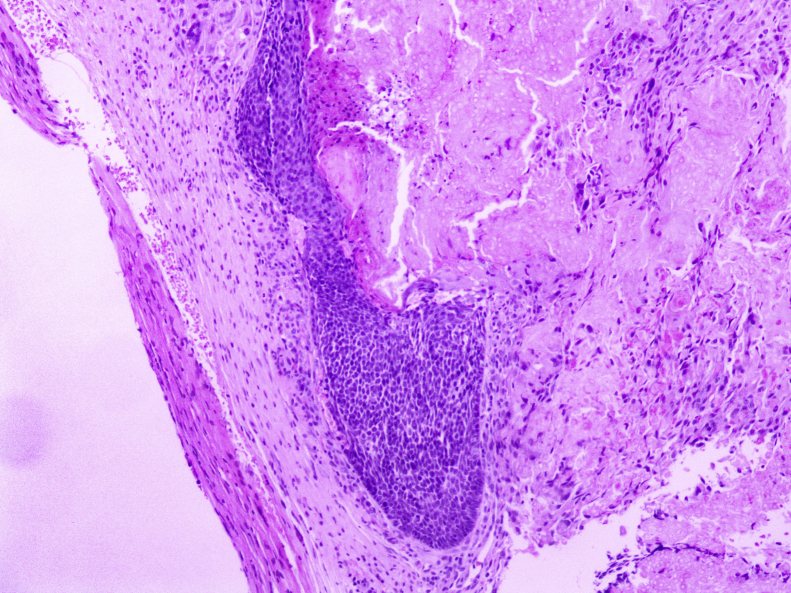
Anatomopathological aspect: nodular proliferation composed of basaloid matrix cells and ghost cells (Hematoxylin & eosin, ×10).

**Figure 4:**
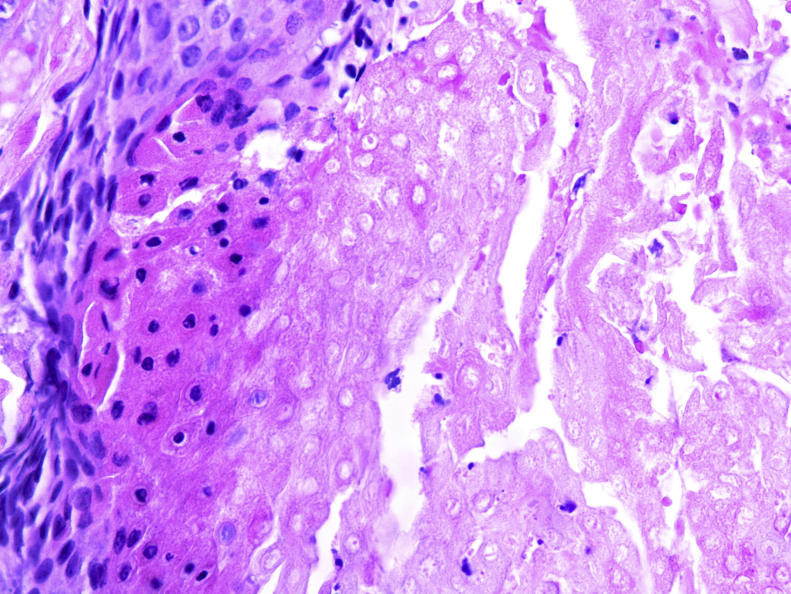
Anatomopathological aspect: nodular proliferation composed of basaloid matrix cells and ghost cells (Hematoxylin & eosin, ×40).

At a dermatological consultation, twin 1 presented a normal, asymptomatic nodular lesion in the left scapular region. An ultrasonography was performed, which showed an echogenic nodular image, emitting a posterior acoustic shadow, measuring 1.4 cm in its largest diameter. In subsequent consultations, over a period of two years, both patients developed multiple similar nodular lesions on the scalp (total of five lesions in twin 1 and four in twin 2). Two of them were excised, one located on the scalp and the other on the scapular region (Fig. 2), both showing histologically a nodular proliferation composed of basaloid matrix cells and phantom cells (Figure 3, Figure 4), findings that were compatible with the diagnosis of pilomatricomas. The remaining lesions were clinically followed-up. The patients had no family history of pilomatricomas.

## Discussion

Pilomatricomas are usually benign solitary lesions. However, these tumors can present as multiple lesions and, although they can occur in healthy individuals, it is recommended to collect a detailed family history and discard associated syndromes.^7^

Myotonic dystrophy and FAP are the syndromes most related to multiple pilomatricomas. The association between RTS and pilomatricomas was first published in 1994^6^ and, since then, eight more cases have been described. However, only five of these cases related multiple pilomatricomas to the syndrome.^2^

A 2019 review of the syndromes associated with multiple pilomatricomas found that non-syndromic patients tend to have fewer pilomatricomas when compared with syndromic patients.^2^ While 4.5% of non-syndromic individuals developed more than five pilomatricomas, 46.3% of syndromic patients developed six or more tumors. However, although the relationship between multiple pilomatricomas and the underlying syndrome strengthens as the number of pilomatricomas increases, some syndromes may have only one or two lesions.^2^ The review of the RTS cases described until 2019 showed that the number of pilomatricomas associated with this syndrome is quite varied. From a total of nine cases, four cases had two to five pilomatricomas, one had more than 10 and four had solitary pilomatricomas.^1^, ^2^, ^5^, ^6^^,^^8^, ^9^, ^10^ Furthermore, a series of four cases published in 1998 showed that, in this group of patients, the mean age of tumor onset was not earlier than in healthy patients, in whom most lesions appear between the ages of 8 months and 10 years.^9^

The etiology of pilomatricoma in RTS has yet to be elucidated. Mutations in two genes, CREBBP and EP300, have been observed in affected individuals and both encode homologous proteins that act as transcription co-activators. During organogenesis, CREBBP is expressed in specific cell types of the developing heart, vasculature, skin, lungs, and liver. In 2016, the first case of RTS with multiple pilomatricomas diagnosed by CREBBP mutation analysis was reported. However, the correlation between the CREBBP genotype and the onset of multiple pilomatricomas still needs to be clarified, since there are also case reports that describe the CREBBP mutation in patients with RTS without pilomatricomas or with a solitary lesion.^5^

Data on pilomatricomas in RTS are still limited to some case reports. In the literature review. This is the first report of multiple pilomatricomas in twins with RTS, reinforcing the association between these two entities. The molecular mechanisms that lead patients with RTS to a greater susceptibility to pilomatricomas warrants further studies.

In some cases, the detection of pilomatricomas may offer an opportunity for the diagnosis of RTS. The therapeutic approach to multiple lesions in RTS is yet to be established. In the patients presented, a conservative approach was chosen, and they are being followed-up without intervention.

## Financial support

None declared.

## Authors’ contributions

Ana Laura Andrade Bueno: Approval of the final version of the manuscript; conception and planning of the study; elaboration and writing of the manuscript; obtaining, analyzing, and interpreting the data; effective participation in research orientation; intellectual participation in propaedeutic and/or therapeutic conduct of studied cases; critical review of the literature; critical review of the manuscript.

Maria Emilia Vieira de Souza: Approval of the final version of the manuscript; conception and planning of the study; elaboration and writing of the manuscript; obtaining, analyzing, and interpreting the data; intellectual participation in propaedeutic and/or therapeutic conduct of studied cases; critical review of the literature; critical review of the manuscript.

Carla Graziadio: Approval of the final version of the manuscript; conception and planning of the study; elaboration and writing of the manuscript; obtaining, analyzing, and interpreting the data; effective participation in research orientation; intellectual participation in propaedeutic and/or therapeutic conduct of studied cases; critical review of the literature; critical review of the manuscript.

Ana Elisa Kiszewski: Approval of the final version of the manuscript; conception and planning of the study; elaboration and writing of the manuscript; obtaining, analyzing, and interpreting the data; effective participation in research orientation; intellectual participation in propaedeutic and/or therapeutic conduct of studied cases; critical review of the literature; critical review of the manuscript.

## Conflicts of interest

Ana Laura Andrade Bueno, Maria Emilia Vieira de Souza, and Carla Graziadio declare to have no conflict of interest. Ana Elisa Kiszewski declares to have a conflict of interest with the company Johnson & Johnson.
